# Improved Microarray-Based Decision Support with Graph Encoded Interactome Data

**DOI:** 10.1371/journal.pone.0010225

**Published:** 2010-04-19

**Authors:** Anneleen Daemen, Marco Signoretto, Olivier Gevaert, Johan A. K. Suykens, Bart De Moor

**Affiliations:** Department of Electrical Engineering ESAT/SCD, Katholieke Universiteit Leuven, Leuven, Belgium; Georgia Institute of Technology, United States of America

## Abstract

In the past, microarray studies have been criticized due to noise and the limited overlap between gene signatures. Prior biological knowledge should therefore be incorporated as side information in models based on gene expression data to improve the accuracy of diagnosis and prognosis in cancer. As prior knowledge, we investigated interaction and pathway information from the human interactome on different aspects of biological systems. By exploiting the properties of kernel methods, relations between genes with similar functions but active in alternative pathways could be incorporated in a support vector machine classifier based on spectral graph theory. Using 10 microarray data sets, we first reduced the number of data sources relevant for multiple cancer types and outcomes. Three sources on metabolic pathway information (KEGG), protein-protein interactions (OPHID) and miRNA-gene targeting (microRNA.org) outperformed the other sources with regard to the considered class of models. Both fixed and adaptive approaches were subsequently considered to combine the three corresponding classifiers. Averaging the predictions of these classifiers performed best and was significantly better than the model based on microarray data only. These results were confirmed on 6 validation microarray sets, with a significantly improved performance in 4 of them. Integrating interactome data thus improves classification of cancer outcome for the investigated microarray technologies and cancer types. Moreover, this strategy can be incorporated in any kernel method or non-linear version of a non-kernel method.

## Introduction

Patients with similar clinical and pathological characteristics such as age, tumor size, lymph node status and grade often differ in clinical outcome and therapy response. Patients for who these traditional diagnostic and prognostic tools fail can potentially be discerned with microarray technology. This technology investigates the transcriptomic make-up of a tumor in one experiment. A decade ago, it was first used in cancer studies to classify tissues as cancerous or non-cancerous [1]–[2] and has since emerged as a popular tool to study different cancer types and outcomes [3]–[6]. Currently within the domain of cancer, microarray technology has earned a prominent place for its capacity to characterize the underlying tumor behavior in detail, leading to an improved diagnostic and prognostic capability. The earliest and most exhaustive efforts have been accomplished for breast cancer [7]. In this manuscript, we aim to improve the predictive power in diagnosis and prognosis of cancer with gene expression data as predictors, by incorporating side information about interactome networks in kernel methods.

**Figure 1:**
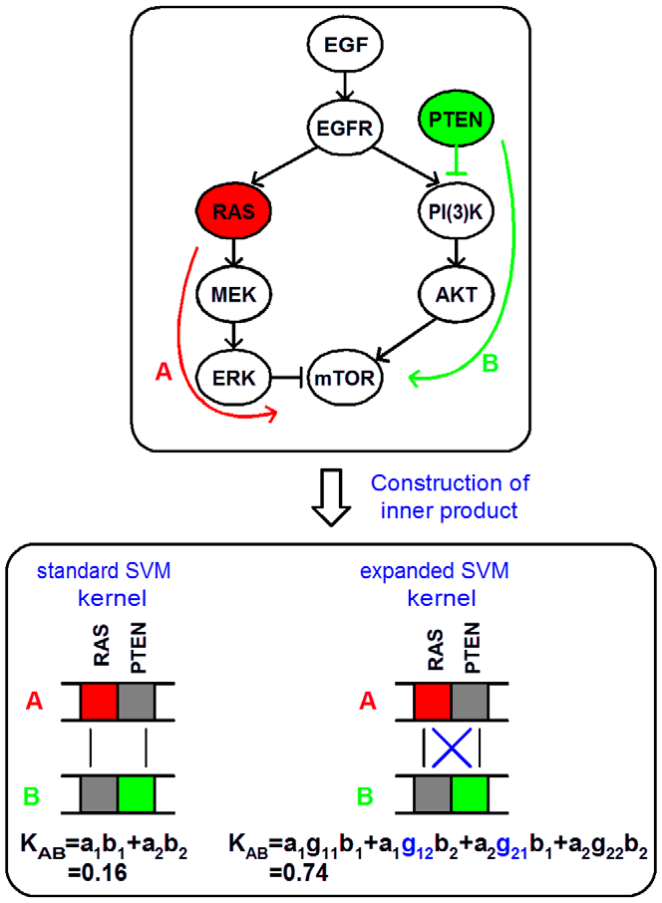
Complementary pathway information and its incorporation in the calculation of patients' similarity. Patients with the same phenotype can be genotypically different since alternative trajectories in a pathway are activated or repressed. Ideally, one wants a classifier that does not make a distinction between alternative pathways. Suppose patients A and B both have breast cancer. However, patient A is characterized by repression of *RAS* while *PTEN* is silenced in patient B [72]. When building a model to distinguish breast cancer patients from control samples, the calculated similarity between patients A and B should be high. When calculating the pairwise gene-product (that is, inner product) between these patients however, they appear to be rather dissimilar with an empirical kernel value of 0.16 due to the different expression for the genes *RAS* and *PTEN*. A more accurate similarity measure would be obtained by considering second order interactions between genes. Genes that are known to interact are assigned a larger weight in the calculation of patients' similarity. Such interactions are first identified based on, for example, shared pathway membership obtained from the KEGG database. This is followed by an exhaustive product between patients' expression profiles that takes gene links into account, weighted according to gene neighborhood in graphs constructed from databases such as KEGG. This will empirically increase the kernel value to 0.74.

In the above mentioned studies, genes were treated as single entities without regard to their neighbors in the interactome network consisting of a wide variety of interaction pairs such as protein-protein (PPI), domain-domain (DDI) and microRNA-mRNA interactions. In Figure 1, a simplified visualization of the mTOR pathway and its regulating pathways is depicted, representing a typical network related to oncogenesis. Several components of these pathways are deregulated in a broad spectrum of human cancers. In this example, the mTOR pathway is switched on either when expression of *RAS* is reduced due to a mutation or when the tumor suppressor gene *PTEN* is inactive. Ideally one wants a classifier that does not make a distinction between both situations. Moreover, the importance of networks instead of individual genes has already been demonstrated by the low gene overlap between prognostic gene signatures in breast cancer but high overlap in relevant pathways [8], with proliferation being the most important common driving force [9]. Genes with similar functions but active in alternative pathways should be taken into account to improve classification performance. Such complementary pathways in which a signal can be propagated through two or more parallel paths have extensively been shown to exist. A well known example shared between the majority of cancer types is *p5*3-mediated apoptosis [10]. A multitude of mechanisms for apoptosis are triggered by the tumor suppressor gene *p53*, among them two distinct apoptotic signaling pathways. Independent on the specific pathway that causes inactivation of *p53*, cancer patients in which apoptosis takes place should be marked as similar with regard to the expression of those genes involved in *p53*-mediated apoptosis. As will be shown, our method exploits such complementary relations between paths by considering second order interactions between genes, available in external databases.

Although gene expression profiles have shown to improve standard tools on clinical and histopathological parameters, the transcriptome is not the only omics layer that reflects the molecular biology of a disease. Many omics layers such as proteomics and metabolomics are interconnected and potentially equally important. We previously presented a kernel-based approach for clinical decision support in which many genome-wide data sources can be combined [11]. Models based on multiple omics layers outperformed the models that were based on one single data set. In the majority of current studies, however, only microarray data are available. In addition, microarray studies have been subject to several criticisms and concerns. Microarray data suffer from a low signal-to-noise ratio, the number of tumor samples used for training and validation are often limited, and gene signatures constructed for similar prediction tasks exhibit very low overlap due to their instability and dependence on the choice of the training samples [12]–[13]. It is therefore useful to expand classification models with gene-related information.

There have been efforts in studying the combined use of gene expression data with biological networks; however, almost all within the gene space for the purpose of functional annotation. Vert and Kanehisa [14] extracted active pathways from gene expression data by using the metabolic network from the KEGG database as prior knowledge. Both the metabolic network and the microarray data were represented as a gene×gene kernel matrix. They were combined in feature space with a generalized form of canonical correlation analysis to learn a semantic representation independent of the two views. Hanisch and colleagues [15] proposed a variant of hierarchical clustering with an increased stability and biological plausibility of the obtained clusters, interpretable as co-regulated pathways. This was obtained by combining a distance function derived from gene expression data and one based on a biological network such as KEGG. Li and Li [16] went one step further by incorporating *a priori* network information into regression analysis for the identification of genes and subnetworks related to diseases or other biological processes. They considered the Laplacian matrix of the network as penalty term in a general regression framework.

These methods all focus on genes, while the aim of our work is to incorporate interactome information to improve classification for cancer patients. Many databases on different aspects of the interactome, of which an exhaustive overview is given in [17], are made freely available to the research community. We will refer to these databases as *secondary data sources*. It would be useful to expand classification models with these sources in the form of the human interactome. PPI networks have recently been introduced to extract subnetworks of interacting proteins or to identify deregulated molecular interactions, evolving from a pathway-based to a protein-network-based approach [18]–[21]. Rapaport and colleagues [22] applied similar ideas to differentiate irradiated from non irradiated yeast strains, by including *a priori* pathway knowledge in the analysis of gene expression data. The high-frequency components in the data were removed with respect to the topology of the gene network, after which the smoothed data was used for classification. Their approach was based on the assumption that low-frequency components in gene expression data contain most biologically relevant information. We, however, will not restrict our analysis to this hypothesis of similar gene expression levels for neighboring genes on the network.

The previously described approaches only focused on one protein-related source. A single source, however, is not necessarily optimal for all cancer-related prediction challenges. In this contribution, we will not limit our strategy solely to pathways or PPIs. Multiple secondary data sources can be extracted from databases, such as KEGG [23], REACTOME [24] and OPHID [25] (for an overview see Table 1). These sources contain gene-related information at other levels of biological regulation than measured with microarray technology. In order to improve microarray-based cancer classification, we investigated how to combine these sources with a patient-based kernel matrix and present a method that is able to incorporate any type of interactome data in the classification process. Where with kernel methods similarity between patients is traditionally calculated with a similarity measure based on the patients' gene expression profiles, interactome data from secondary data sources can be used to improve the method how patient similarities are calculated. At the same time, we hypothesize to improve classification performance. This is motivated by the principle that in patients within the same cancer-subgroup, different genes from the same pathway can be expressed, making single-gene markers not ideal (see Figure 1). Furthermore, because the relevance of the databases for each specific problem is not known beforehand and irrelevant databases may worsen the results, we present multiple well-considered schemes for combining the information from secondary data sources.

**Table 1:** Secondary data sources.

	secondary data source	type of pathway resource	definition gene pairs	# gene pairs	release	reference
1	KEGG	metabolic pathways	genes of which the proteins belong to the same pathway	609.269	49.0	[23]
2	HumanCyc	metabolic pathways	genes of which the proteins belong to the same pathway	12.314	12.0	[39], [40]
3	EHMN	metabolic pathways	genes of which the proteins belong to the same pathway	198.876	/	[38]
4	REACTOME	metabolic pathways, signaling pathways	genes involved in the same reaction or complex	722.508	28	[24]
5	OPHID	protein-protein interactions, genetic interaction networks	genes of which the proteins interact	221.674	1.71	[25]
6	BioGRID	protein-protein interactions	genes of which the proteins interact	40.812	2.0.54	[42]
7	STRING	protein-protein interactions	experimentally determined gene interactions	315.686	8.1	[48]
8	DOMINE	domain-domain interactions	genes with proteins interacting via a domain-domain interaction	18.213.973	1.1	[49]
9	UniDomInt	domain-domain interactions	genes with proteins interacting via a domain-domain interaction	20.506.327	Aug '09	[50]
10	PROSITE	protein families and domains	genes with one or multiple protein domains, families or functional sites in common	6.267.453	20.0	[51]
11	Pfam	protein families and domains	genes with one or multiple protein domains or families in common	3.649.554	23.0	[52]
12	miRBase	transcription factors, gene regulatory networks	genes targeted by the same miRNA	6.819.380	5	[53]
13	miRNAmap	transcription factors, gene regulatory networks	genes targeted by the same miRNA	28.120.039	2	[54]
14	microRNA.org	transcription factors, gene regulatory networks	genes targeted by the same miRNA	13.207.828	Sept '08	[56]
15	TargetScan	transcription factors, gene regulatory networks	genes targeted by the same miRNA	8.580.619	5.1	[57]

## Results

Kernel Methods, a powerful class of methods for pattern analysis, have become a standard tool in data analysis, computational statistics and machine learning applications due to their reliability, accuracy and computational efficiency [26]. Although the idea of secondary data incorporation is applicable to any kernel method or method that can be kernelized, we present results for the weighted Least Squares Support Vector Machine (LS-SVM), a method for supervised classification that takes the typical unbalance in many two-class problems into account [27]–[29].

### The considered microarray data sets

For this contribution, we mainly focused on breast cancer because it is one of the most extensively studied cancer types for which many microarray data sets are publicly available. In addition, data sets on ovarian cancer, prostate cancer and diffuse large-B-cell lymphoma were included. Table 2 gives an overview of the 16 studied data sets with information on outcome, microarray platform and number of included samples and genes. These studies cover a wide range of predictable, cancer-related outcomes such as response, relapse, metastasis and survival. We took into account the low signal-to-noise ratio of microarray data and included the 5000 most varying genes (see materials and methods section).

**Table 2:** Microarray data sets.

	Data set	cancer type	Outcome	Platform	# samples (neg/pos)	# genes
T1	Berchuck [5]	OC	Binary survival (short< = 3 yrs vs. long >7 yrs)	U133a	53 (29/24)	12633
V1	Bild [33]	OC	Binary survival (short< = 3 yrs vs. long >3 yrs)	U133a	133 (88/45)	11911
V2	Chin [6]	BC	Distant recurrence (no vs. yes)	U133av2	129 (102/27)	12633
T2	Hess [73]	BC	Pathologic response (RD vs. CR)	U133a	133 (99/34)	12633
V3	Huang 1 [4]	BC	Disease recurrence (no vs. yes)	U95av2	52 (34/18)	8740
V4	Huang 2 [4]	BC	Relapse (no vs. yes)	U95av2	80 (53/27)	8740
T3	Ivshina [74]	BC	Local, regional or distant recurrence (no vs. yes)	U133a+b	249 (160/89)	18001
V5	Miller [75]	BC	Death from BC (no vs. yes)	U133a+b	236 (181/55)	18001
T4	Pittman 1 [76]	BC	Relapse (no vs. yes)	U95av2	158 (95/63)	8740
V6	Pittman 2 [76]	BC	Loco-regional recurrence (no vs. yes)	U95av2	158 (132/26)	8740
T5	Pittman 3 [76]	BC	Distant metastasis (no vs. yes)	U95av2	158 (108/50)	8740
T6	Rosenwald [34]	DLBCL	Overall survival (short<4 yrs vs. long > = 4 yrs)	Lymphochip	220 (118/102)	6707
T7	Singh [3]	PC	Tumor status (normal vs. tumor)	U95av2	102 (50/52)	8193
T8	Sotiriou 1 [77]	BC	Relapse (no vs. yes)	U133a	187 (120/67)	12633
T9	Sotiriou 2 [77]	BC	Distant metastasis (no vs. yes)	U133a	179 (139/40)	12633
T10	Wang [35]	BC	Metastasis within 5 yrs (no vs. yes)	U133a	276 (183/93)	11911

T, training data set; V, validation data set.

BC, breast cancer; OC, ovarian cancer; DLBCL, diffuse large-B-cell lymphoma; PC, prostate cancer.

RD, residual disease; CR, complete response.

### Interactome data as prior biological knowledge

The human interactome is the compendium of all stable, transient, direct and indirect physical interactions between proteins in molecular machines and pathways in an active cell. For cancer, much biological knowledge and pathway information is available in databases on different aspects of biological systems [17]. A distinction can be made between protein-protein interactions (PPI), domain-domain interactions (DDI), metabolic pathways, signaling pathways, transcription factors, gene regulatory networks and protein-compound interactions. Table 1 gives an overview of the 15 considered secondary data sources, with the type of pathway information contained in each of them, the definition of links between genes, and the number of extracted gene pairs. A more detailed description can be found in the materials and methods section.

### Representation of interactome data based on spectral graph theory

**Figure 2:**
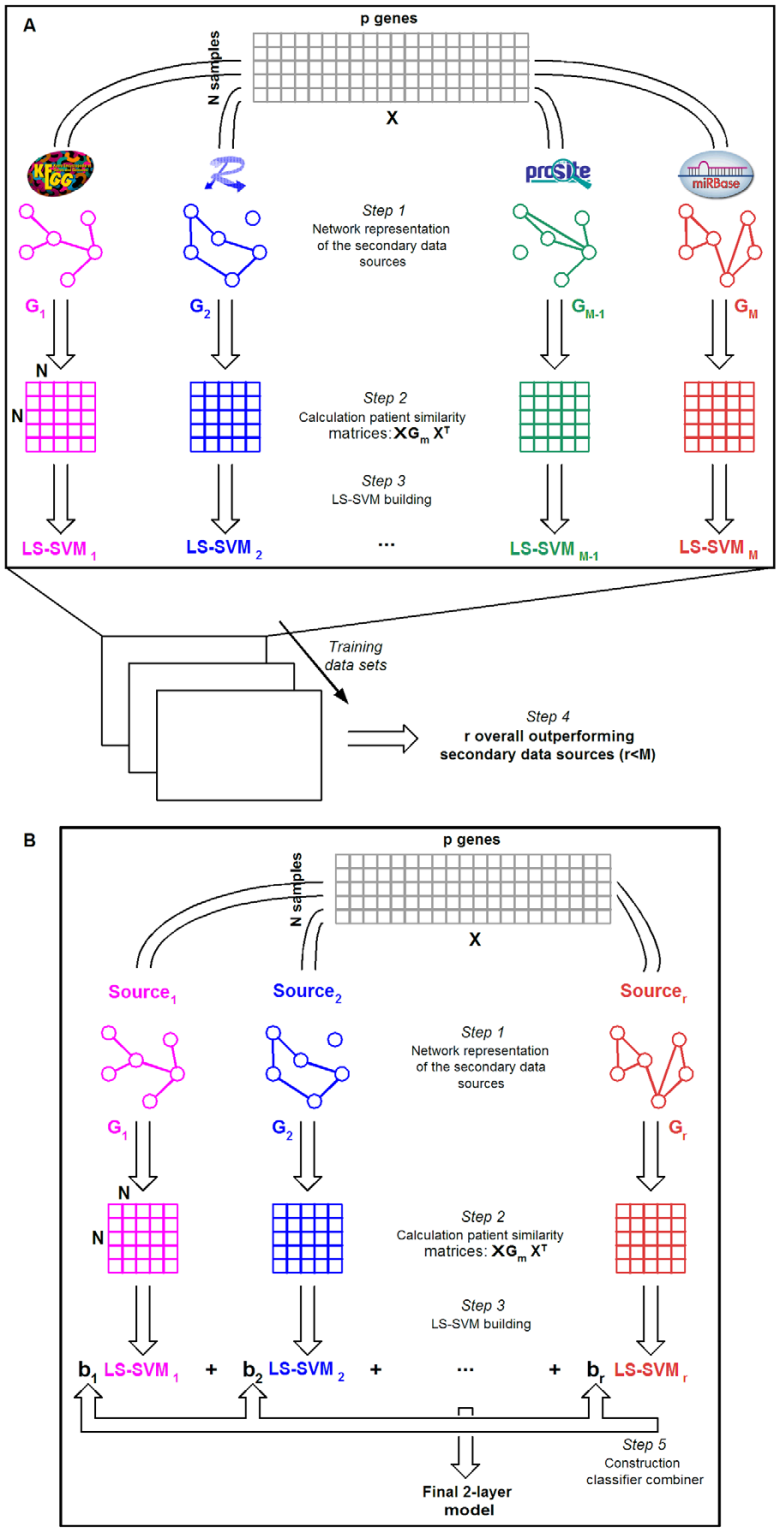
Global overview of the methodology. (**A**) In step 1, *M* secondary data sources are represented as a graph. The graph-related information is subsequently incorporated via the pseudoinverse of the Laplacian (the *G*-matrix) into the calculation of patient similarity. In step 3, LS-SVM models are built on each of the updated kernel matrices obtained in step 2. Based on a set of microarray training data, out of *M* secondary data sources, *r* sources are selected that increase performance for all training data sets with respect to models built on only microarray data. (**B**) After having selected the *r* outperforming secondary data sources, steps 1 to 3 are repeated for those *r* sources. In step 5, a classifier is learned and constructed for the combination of the *r* corresponding LS-SVM models.

To incorporate the previous described secondary data sources in a kernel framework for microarray-based cancer classification presented in Figure 2, the databases were converted into graphs from which the corresponding Laplacian matrix can be derived (step 1 in Figure 2). The pseudoinverse of the Laplacian [30], from now on referred to as *G*-matrix, was used to represent similarity between pairs of genes. With this graph-based approach, both direct and indirect connections between genes, and thus their neighborhood in the human interactome are taken into account. Genes that do not belong to the same pathway but are connected to a same subset of genes, for example, are assigned a positive coefficient in the secondary *G*-matrix. These matrices were incorporated in the calculation of patient similarity (step 2 in Figure 2). This corresponds to replacing the standard inner product by a quadratic form defined upon *G^−1^* (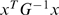), and is interpretable as a weighted version of the standard inner product. An example is presented in Figure 1.

To get an idea about the extra information that is added by the secondary data sources with respect to the standard inner product, Table 3 shows the median (25^th^–75^th^ percentile) number of second order interactions included in each of the 15 secondary data sources. These numbers slightly vary per source as the 5000 most varying genes selected from each microarray data set differ. The larger amount of second order interactions in the *G*-matrices with respect to the Laplacian matrices shows that the network neighborhood is captured by taking the pseudoinverse of the Laplacian.

**Table 3:** Characteristics of the Laplacian and *G*-matrices.

	Secondary data source	Laplacian: median # gene pairs	Laplacian: 25^th^–75^th^ percentile	*G*-matrices: median # gene pairs	*G*-matrices: 25^th^–75^th^ percentile
1	KEGG	69.870	41.771–99.310	5.553.761	4.950.232–6.473.276
2	HumanCyc	533	298–752	4.616.183	4.548.199–5.524.069
3	EHMN	5.416	3.339–11.847	9.356.394	8.954.741–10.040.781
4	REACTOME	43.611	27.716–59.786	6.453.263	4.585.327–7.725.010
5	OPHID	5.261	2.783–9.601	10.821.416	9.713.765–11.068.831
6	BioGRID	1.995	1.067–3.613	11.047.443	9.744.897–11.811.681
7	STRING	4.327	2.531–8.512	10.563.906	10.331.629–10.811.510
8	DOMINE	744.622	568.318–878.721	11.221.950	10.719.122–11.353.931
9	UniDomInt	863.228	667.232–1.019.495	11.541.609	10.635.969–11.826.811
10	PROSITE	256.028	221.618–276.509	10.967.586	10.962.602–11.560.607
11	Pfam	145.582	112.127–153.525	12.199.330	12.014.816–12.258.684
12	miRBase	342.756	275.376–347.322	10.136.253	9.243.720–10.163.274
13	miRNAmap	430.310	237.475–600.733	2.554.930	1.517.502–3.324.331
14	microRNA.org	228.423	148.857–350.045	2.025.078	1.410.494–2.800.161
15	TargetScan	422.876	242.981–656.470	5.420.278	3.766.078–6.798.828

### Selection of outstanding secondary data sources

To reduce the set of considered secondary data sources to the ones that are relevant across multiple cancer types and usable for multiple outcomes, 10 out of 16 microarray data sets were randomly selected for training, indicated in Table 2. This reduction in number of secondary data sources is represented in Figure 2 as step 4. For the 10 training data sets, the data were randomly split into 10 folds. Five folds were used for model building based on each individual secondary data source, while 2 folds served for validation (the remaining 3 folds are used later for the combination of multiple models). To obtain a good estimate for the generalization error, this procedure was repeated 200 times. To objectively select those secondary data sources that performed well for the training microarray data sets, models built on an individual secondary data source with a better average performance than the baseline model (without inclusion of secondary data) were given a decreasing score starting from 15 for the best model. Models that performed worse than baseline were given the score 0. Adding those scores for each secondary data source over all training sets highlighted three outstanding secondary data sources: KEGG, OPHID and microRNA.org (see Table 4). Only these three databases were used for the remaining data sets.

**Table 4:** Selection of secondary data sources based on the training microarray data sets.

	1	2	3	4	5	6	7	8	9	10	11	12	13	14	15
**T1**	14	9	0	11	10	0	0	13	15	12	0	8	0	0	0
**T2**	0	0	0	0	14	0	15	0	0	0	0	0	0	0	0
**T3**	0	0	0	14	15	0	0	0	0	0	12	0	11	13	10
**T4**	15	0	0	0	11	0	13	0	0	0	0	0	12	14	0
**T5**	15	0	0	0	0	0	11	0	0	0	0	0	14	12	13
**T6**	14	13	0	0	15	0	0	0	0	0	0	0	12	0	0
**T7**	15	0	0	14	0	0	0	0	0	0	0	0	0	13	0
**T8**	0	13	0	0	0	0	0	0	0	0	0	0	0	14	15
**T9**	7	0	12	0	15	9	10	14	0	11	0	0	0	13	8
**T10**	0	0	0	0	12	0	0	0	0	15	0	0	14	13	0
**Sum**	**80**	35	12	39	**92**	9	49	27	15	38	12	8	63	**92**	46

Individual models with a better performance than the baseline model are given a decreasing score starting from 15 for the best model; models that perform worse are given the score 0.

The mutual information (MI) between the models based on one of the three selected secondary data sources was on average 0.376, varying from 0.174 to 0.607 for the 10 training data sets and indicating certain degree of independence between these sources. No relationship was found between MI and the increase in performance with respect to baseline. Seven of the 10 training microarray data sets are from breast cancer (see Table 2). However, as can be seen from Table 4, the increase in performance caused by the three selected secondary data sources was not limited to breast cancer. KEGG performed well for all cancer types, OPHID resulted in a better performance for breast cancer, ovarian cancer and lymphoma, while microRNA.org led to an increased performance for both breast and prostate cancer. Based on the training data sets, the data sources STRING and TargetScan seemed to be specific to breast cancer. Finally, there were no databases that simultaneously led to an improved performance for the same training data sets.

### Combination of multiple classifiers

Because the relevance of these three sources for each specific classification task is not known beforehand, the three corresponding individual classifiers were combined at a second level (step 5 in Figure 2). Three types of combination rules were considered: 1) fixed rules for which no training is required (that is, mean and median), 2) simple trained rules for which the influence of each model on the final prediction is determined by their individual training performance or for which the optimal combination of individual models is obtained with an exhaustive search, and 3) more advanced models, being naïve Bayes, logistic regression and linear discriminant analysis. For each of the 200 experiments, the last two types of combination rules were trained on 8 folds (that is, including the 5 folds upon which each model was built expanded with 3 untouched folds) and validated on the remaining 2 folds. The global workflow consisting of the graph representation of the secondary data sources, the incorporation into the calculation of patient similarities, model building, the selection of the relevant secondary data sources, the combination of relevant models and validation is provided in Figure 2.

### Incorporation of the three outstanding secondary data sources outperforms the baseline models

**Figure 3:**
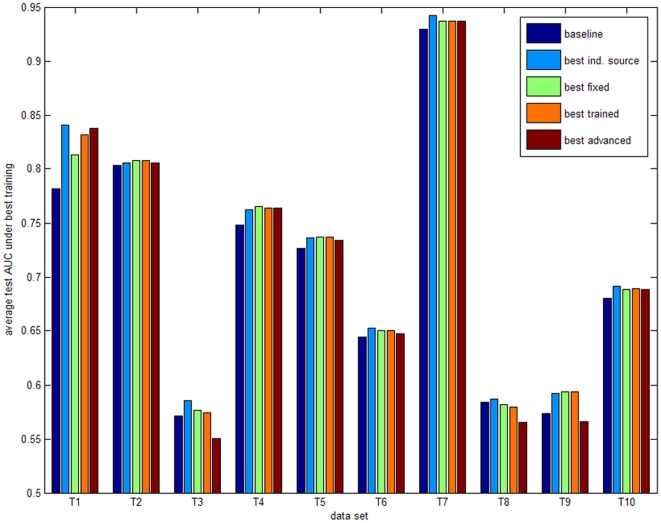
Overall training results. Influence of secondary data sources and classifier combination on classification performance. The average test AUC values under best training for 10 training microarray data sets, referred to as T1 to T10 are shown. Per training set, five bars are shown: 1) mean AUC of the baseline model (blue); 2) mean AUC for the best individual secondary data source (KEGG, OPHID or microRNA.org) (cyan); 3) mean AUC for the best fixed combination rule (green); 4) mean AUC for the best trained combination rule (orange); 5) mean AUC for the best advanced model (brown). The secondary data source and combination rules that performed best and the p-values for the comparisons per training set are shown in Table 5.

**Figure 4:**
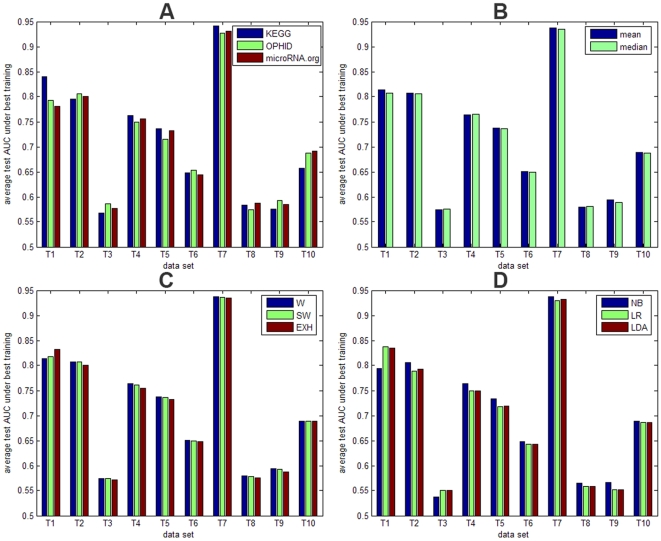
Training results per type of combination rule. Average test AUC values under best training for classifiers or combined classifiers, each built on one of the outstanding secondary data sources KEGG, OPHID or microRNA.org for 10 training microarray data sets. (**A**) mean AUC values for the individual classifiers, built on KEGG (blue), OPHID (green) or microRNA.org (brown); (**B**) mean AUC values for two fixed rules: mean (blue) and median (green); (**C**) mean AUC values for three trained rules: weighted with the 5-fold training AUC values (W) (blue), weighted with the 5-fold training AUC values scaled to ]0,1] (SW) (green) and exhaustive search among all possible combinations of 3 classifiers (EXH) (brown); (**D**) mean AUC values for three more advanced models: naïve Bayes (NB) (blue), logistic regression (LR) (green) and linear discriminant analysis (LDA) (brown).

The results for the 10 training microarray data sets are shown in Figure 3 and Table 5. The five bars per data set represent the mean area under the receiver operating characteristic curve (AUC) values for the following models: 1) the baseline model built on the microarray data only, 2) the model based on the secondary data source with the best performance (KEGG, OPHID or microRNA.org), 3) the combination of models according to the best fixed rule, 4) the combination according to the best trained rule, and 5) the combination of individual models using the best advanced approach. Figure 4a provides an overview of the performance of the three selected secondary data sources for all training data sets. As the prediction accuracy is evaluated by applying our 10-fold approach 200 times, the 200 test AUC values of the baseline model were compared with the 200 test AUC values of the other considered models using the one-sided paired-sampled t-test. The p-values for these comparisons are shown per data set in Table 5. These results have been confirmed by applying the Wilcoxon signed-ranks test to the average performance of all data sets. The model based on the best individual secondary data source outperformed the baseline models over all training data sets (p-value 0.002). Also combining classifiers based on individual secondary data sources with a fixed or trained rule outperformed the baseline models with a p-value of 0.0039 and 0.0098, respectively. The more advanced models did not perform unambiguously better (p-value 0.557), although an improvement was observed when reducing the number of classifiers.

**Table 5:** Influence of secondary data sources and classifier combination on the performance for 10 training microarray data sets.

Data set	model (combination)	source/rule§	mean AUC (std)*	p-value°	−log(p)
T1	baseline		0.782 (0.113)		
	best individual	KEGG	0.840 (0.100)	1.51e-25	24.82
	best fixed rule	mean	0.813 (0.105)	2.00e-17	16.70
	best trained rule	EXH	0.832 (0.104)	1.28e-20	19.89
	best advanced rule	LR	0.838 (0.105)	3.39e-12	11.47
T2	baseline		0.803 (0.081)		
	best individual	OPHID	0.805 (0.078)	0.199	0.70
	best fixed rule	mean	0.808 (0.081)	0.0113	1.95
	best trained rule	W	0.808 (0.081)	0.0114	1.94
	best advanced rule	NB	0.806 (0.081)	0.125	0.90
T3	baseline		0.571 (0.073)		
	best individual	OPHID	0.586 (0.078)	6.11e-5	4.21
	best fixed rule	median	0.576 (0.076)	0.0121	1.92
	best trained rule	W	0.575 (0.077)	0.089	1.05
	best advanced rule	LDA	0.550 (0.085)	0.999	0.00
T4	baseline		0.748 (0.088)		
	best individual	KEGG	0.762 (0.085)	8.17e-7	6.09
	best fixed rule	median	0.765 (0.086)	7.30e-15	14.14
	best trained rule	W	0.764 (0.085)	1.87e-15	14.73
	best advanced rule	NB	0.764 (0.087)	3.40e-15	14.47
T5	baseline		0.727 (0.089)		
	best individual	KEGG	0.736 (0.091)	0.000437	3.36
	best fixed rule	mean	0.737 (0.090)	6.02e-6	5.22
	best trained rule	W	0.737 (0.090)	8.77e-6	5.06
	best advanced rule	NB	0.734 (0.090)	0.000644	3.19
T6	baseline		0.645 (0.078)		
	best individual	OPHID	0.653 (0.077)	9.73e-9	8.01
	best fixed rule	mean	0.650 (0.078)	2.74e-7	6.56
	best trained rule	W	0.650 (0.078)	3.27e-7	6.48
	best advanced rule	NB	0.647 (0.077)	0.0747	1.13
T7	baseline		0.929 (0.056)		
	best individual	KEGG	0.942 (0.048)	6.02e-10	9.22
	best fixed rule	mean	0.937 (0.050)	2.27e-11	10.64
	best trained rule	W	0.937 (0.050)	3.45e-11	10.46
	best advanced rule	NB	0.937 (0.050)	1.36e-10	9.87
T8	baseline		0.584 (0.096)		
	best individual	microRNA.org	0.587 (0.096)	0.156	0.81
	best fixed rule	median	0.581 (0.096)	0.785	0.10
	best trained rule	W	0.579 (0.093)	0.943	0.03
	best advanced rule	NB	0.565 (0.096)	0.999	0.00
T9	baseline		0.573 (0.110)		
	best individual	OPHID	0.592 (0.117)	0.0151	1.82
	best fixed rule	mean	0.594 (0.113)	0.00842	2.07
	best trained rule	W	0.594 (0.113)	0.00787	2.10
	best advanced rule	NB	0.566 (0.117)	0.797	0.10
T10	baseline		0.680 (0.070)		
	best individual	microRNA.org	0.691 (0.065)	0.0176	1.75
	best fixed rule	mean	0.688 (0.065)	0.0596	1.23
	best trained rule	SW	0.689 (0.065)	0.0419	1.38
	best advanced rule	NB	0.688 (0.066)	0.0586	1.23

§secondary data source or combination rule with the best performance (W = weighting according to the 5-fold training AUC values; SW = weighting with scaled 5-fold training AUC values; EXH = exhaustive search among all possible combinations of 3 classifiers; NB = naïve Bayes; LR = logistic regression; LDA = linear discriminant analysis).

*mean test AUC value under best training (standard deviation).

°one-sided paired-sampled t-test for the comparison with respect to the baseline model.

**Figure 5:**
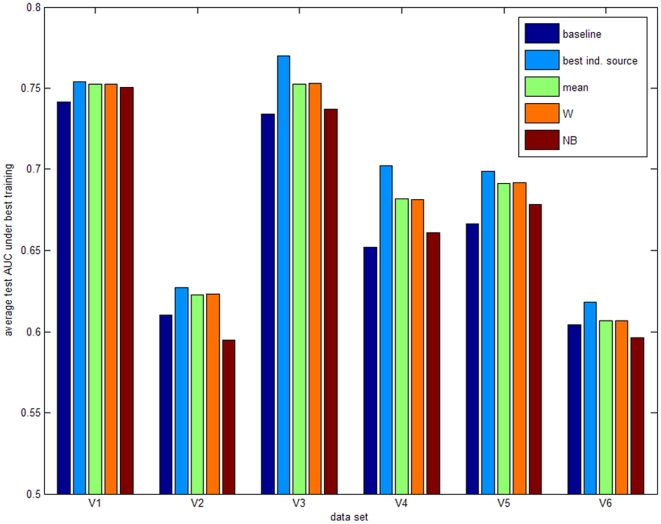
Validation results for the optimal combination rules. Validation of the three selected secondary data sources and the best performing combination rules on six new microarray data sets, referred to as V1 to V6. Per validation data set, five bars with the average test AUC under best training are shown: 1) mean AUC of the baseline model (blue); 2) mean AUC for the best individual secondary data source (KEGG, OPHID or microRNA.org) (cyan); 3) mean AUC for the fixed mean rule (green); 4) mean AUC for the weighted combination rule (orange); 5) mean AUC for the naïve Bayes rule (brown). A numerical overview is given in Table 6.

To define the most optimal combination rules, we compared the average performances for the fixed, trained and advanced combination rules in Figures 4b to 4d, respectively. Based on the training data sets, the mean fixed rule, the weighted trained rule and naïve Bayes performed best. Only these three rules were therefore validated on the remaining 6 microarray data sets. Figure 5 and Table 6 show the validation results when considering KEGG, OPHID and microRNA.org as secondary data sources. The validation data sets confirm the training results: combining the three individual models significantly improved classification in 4 out of 6 data sets, while no significant improvement was obtained in 2 out of 6 data sets. Overall, the best individual secondary data source, the mean fixed rule and the weighted trained rule outperformed the baseline model with a p-value of 0.0313, 0.0313 and 0.0313, respectively. These higher p-values compared to the results on the training data are due to the lower number of validation data sets. When applying the Wilcoxon signed-ranks test to all 16 considered data sets, the p-values decreased to 0.0004, 0.0005 and 0.001, respectively. We can therefore conclude based on the training and validation data sets that averaging the predictions of the three classifiers performs best. Weighting the predictions according to the training AUC values does not provide additional value. The benefit of incorporating secondary data sources was largest for data sets T1, V3 and V4 due to weaker experimental data caused by the limited number of samples.

**Table 6:** Performance of the three selected secondary data sources (KEGG, OPHID, microRNA.org) and the combination rule per type that performed best on the training data (mean, AUC weighting (W) and naïve Bayes (NB)) for 6 validation microarray data sets.

data set	source/rule	mean AUC (std)	p-value°	−log(p)
V1	baseline	0.741 (0.099)		
	KEGG	0.739 (0.097)	0.807	0.09
	OPHID	0.734 (0.097)	0.982	0.01
	microRNA.org	0.754 (0.101)	1.18e-7	6.93
	mean	0.753 (0.096)	1.37e-7	6.86
	W	0.752 (0.096)	3.13e-7	6.50
	NB	0.750 (0.096)	3.37e-6	5.47
V2	baseline	0.610 (0.171)		
	KEGG	0.593 (0.154)	0.899	0.05
	OPHID	0.627 (0.146)	0.0928	1.03
	microRNA.org	0.610 (0.151)	0.509	0.29
	mean	0.623 (0.147)	0.160	0.80
	W	0.623 (0.148)	0.157	0.80
	NB	0.595 (0.157)	0.864	0.06
V3	baseline	0.734 (0.225)		
	KEGG	0.770 (0.220)	2.27e-10	9.64
	OPHID	0.723 (0.230)	0.979	0.01
	microRNA.org	0.749 (0.224)	4.40e-4	3.36
	mean	0.753 (0.225)	4.91e-6	5.31
	W	0.753 (0.225)	5.10e-6	5.29
	NB	0.737 (0.234)	0.380	0.42
V4	baseline	0.652 (0.189)		
	KEGG	0.688 (0.183)	5.11e-9	8.29
	OPHID	0.630 (0.195)	0.999	0.00
	microRNA.org	0.702 (0.180)	4.45e-17	16.35
	Mean	0.682 (0.182)	4.55e-9	8.34
	W	0.682 (0.182)	6.12e-9	8.21
	NB	0.661 (0.182)	0.112	0.95
V5	baseline	0.667 (0.186)		
	KEGG	0.679 (0.180)	0.0128	1.89
	OPHID	0.699 (0.176)	1.75e-6	5.76
	microRNA.org	0.669 (0.187)	0.338	0.47
	Mean	0.691 (0.180)	2.95e-7	6.53
	W	0.692 (0.179)	1.55e-7	6.81
	NB	0.678 (0.187)	0.0428	1.37
V6	baseline	0.604 (0.110)		
	KEGG	0.603 (0.109)	0.656	0.18
	OPHID	0.618 (0.107)	0.00143	2.85
	microRNA.org	0.611 (0.109)	0.00398	2.40
	mean	0.607 (0.106)	0.196	0.71
	W	0.607 (0.106)	0.185	0.73
	NB	0.596 (0.114)	0.949	0.02

°one-sided paired-sampled t-test for the comparison with respect to the baseline model.

## Discussion

In this manuscript, improved outcome prediction and classification decision making have been made possible by incorporating the human interactome in an LS-SVM model, chosen among a large set of possible methods in which prior information can be incorporated in the presented manner. Interactome data from secondary data sources were encoded in a graph-based way and used in similarity matrices for patient classification. Ten microarray data sets were randomly selected for training. For the majority of these data sets, three secondary data sources increased performance with respect to the baseline model based on microarray data only, being KEGG, OPHID and microRNA.org. The 15 secondary databases considered in this study could therefore be reduced to three gene-related information sources that are relevant across multiple cancer types with regard to the considered class of models. These sources also showed a good performance on 6 validation microarray data sets. With only three models to combine, it could be shown on both training and validation data sets that averaging the predictions of the individual models suffices. The outstanding results of equally weighting the individual models are in line with the findings of Lewis and colleagues on kernel matrices [31]. They showed that for many applications, a naive unweighted sum of matrices is sufficient unless multiple noisy data sets are among the available data sets, and that optimization of the weights is only beneficial when sufficient data are available to more reliably estimate the weights. Combination rules that require additional training are therefore only expected to gain in importance when the sample size in microarray studies increases.

The three selected sources KEGG, OPHID and microRNA.org are the most complete databases for their type of information. With the KEGG database, information about groups of genes involved in the same metabolic pathways, pathways related to genetic information processing, regulatory pathways or pathways active in human diseases and drug development, is incorporated. OPHID is a database on predicted interactions between human proteins combining PPIs obtained from specialist literature, high-throughput experiments, evolutionary conservation and other databases such as BIND, HPRD and MINT. In the microRNA.org database, predicted miRNA target sites are filtered according to evolutionary conservation. This database was represented as a network with links between genes that are targeted by the same miRNA. Lu and colleagues [32] observed a general down-regulation of miRNAs in tumors compared with normal tissues. They were able to successfully classify poorly differentiated tumors using miRNA expression profiles. The gene expression profiles lacked this information when applied to the same samples. An improved classification performance by including the microRNA.org network in this study confirms the importance of miRNAs in cancer and their impact on target genes.

The extra layer for the combination of classifiers is essential. For verification, the largest network per type of gene-based information, being KEGG, REACTOME, OPHID, STRING, PROSITE and microRNA.org were pooled (that is, the edge weights were added) before an LS-SVM model was built. However, in all cases our 2-layer approach outperformed models built on a union of secondary data sources. This emphasizes the specific edge interpretation for each type of interaction and makes the construction of individual models per secondary data source essential before combining them at a second level.

In conclusion, we showed that it is possible to incorporate prior information from secondary data sources in the form of the human interactome in any kernel method or non-linear, kernel-based extension of a non-kernel method. Any type of gene-related information can be considered and the matrices derived from each graph are representable in many different ways. Moreover, no part of the data needs to be discarded. All genes are considered, also the ones that have not been thoroughly investigated or for which no annotation is available. Our results show that for the considered microarray technologies, cancer types and types of outcome, integrating interactome data improves classification of cancer outcome based on microarray data. This integration of prior information in an SVM model based on gene expression may benefit investigation of biological functionality.

## Materials and Methods

### Microarray data sets

An overview of the microarray data sets on breast, ovarian, prostate cancer and large-B-cell lymphoma including at least 50 samples is provided in Table 2. For the data set of Bild [33], no binary outcome was available. The median survival time of 3 years was chosen as cut-off to balance both classes of samples. For the data set of Rosenwald [34], the suggested 4 years was taken while in the study by Wang [35], metastasis within 5 years was studied. For these data sets, censored samples with last follow-up before the chosen threshold (3, 4 or 5 years, respectively) were excluded, resulting in the loss of 14, 20 and 10 samples, respectively.

The data sets gathered with the Affymetrix microarray technology were preprocessed with MAS 5.0, the GeneChip Microarray Analysis Suite 5.0 software (Affymetrix). An updated array annotation was used for the conversion of probes to Entrez Gene Ids [36]. A custom-designed microarray composed of genes of which the products are preferentially expressed in lymphoid cells was used in [34]. The data at the Entrez Gene level as provided by the authors were therefore used. Missing gene expression values in this data set were imputed unsupervised using the k-nearest neighbors method [37], reducing the number of genes from 7399 to 6707. The parameter k was set to 15 such that a missing value for a spot S in a sample was estimated as the weighted average of the 15 spots that are most similar to spot S in the remaining samples. We also took the low signal-to-noise ratio of microarray data into account by unsupervised exclusion of genes with low variation. The 5000 most varying genes were retained. Limiting the number of genes to 5000 has the additional advantage that the computation of the pseudoinverse Laplacian matrix remains tractable (see section on the pseudoinverse Laplacian). But then again, the analysis should be performed on a sufficient number of genes between which interactions are described in secondary data sources.

### Description secondary data sources

#### Metabolic pathways

Metabolic pathways were extracted from four databases as each database may contain false positive pathway predictions. Moreover, because most metabolic reactions are linked with disease genes, the human metabolic network is rather fragile [38]. The data source KEGG [23] stands for Kyoto Encyclopedia of Genes and Genomes and is a knowledge base for the analysis of gene functions in terms of networks of genes and molecules. We focused on the KEGG biochemical pathway database containing metabolic pathways, pathways related to genetic information processing, regulatory pathways involved in environmental information processing and cellular processes, and pathways active in human diseases and drug development. As the pathway information is matched with the KEGG gene database, individual genes can be linked to components of KEGG biochemical pathways. Based on these cross-references, we extracted gene pairs defined as genes encoding for proteins that can catalyze two reactions in the same pathway.

Another large repository on metabolic pathways is BioCyc, a collection of more than 350 organism-specific pathway/genome databases (PGDBs) for most eukaryotic and prokaryotic species with sequenced genomes [39]. Every BioCyc PGDB contains the predicted metabolic network for one organism, including metabolic pathways, enzymes, metabolites and reactions. These databases can be divided into three groups, according to the amount of manual curation. In this manuscript, we considered two PGDBs, the intensively curated database MetaCyc [39] and the computationally-derived database HumanCyc, subject to a moderate curation of less than 1 year [40]. HumanCyc contains information on 28783 human genes, their products and the metabolic reactions and pathways they catalyze. The metabolic pathways were predicted based on genome annotation, with missing enzymes within the predicted pathways replaced by candidate proteins when possible. MetaCyc [39], the Multiorganism database of Metabolic Pathways and Enzymes provides a high-quality resource on small-molecule metabolisms and contains experimentally verified metabolic pathway and enzyme information curated from specialist literature. We extracted the human pathways from MetaCyc; however, only one metabolic pathway was not included in HumanCyc. Gene pairs were defined as genes with proteins belonging to the same metabolic pathway, in analogy to KEGG.

Because not all known human metabolic pathways are included in HumanCyc and KEGG, we also considered a more recent human metabolic network, manually reconstructed by integrating genome annotation information from different databases and metabolic reaction information from specialist literature [38]. This network, referred to as EHMN (Edinburgh human metabolic network) contains more than 2000 metabolic genes and almost 3000 metabolic reactions, reorganized into 70 human-specific metabolic pathways. Also here, gene pairs were defined as genes encoding for enzymes that are involved in the same reaction.

The fourth secondary data source is REACTOME [24], an expert-authored knowledge base of human biological processes. The database consists of 2907 reactions involving 2975 human proteins and grouped into pathways taking their temporal relationships and interdependencies into account. It represents pathways of intermediary metabolism, regulatory pathways, signal transduction and high-level processes such as the cell cycle. Gene pairs were defined as genes involved in the same reaction or complex.

#### Protein-protein interactions

Next, several major databases on PPIs are available. PPIs are typically extracted from publications in which high-throughput proteomic or small-scale biomolecular methods were applied. Especially high-throughput methods make PPI networks noisy with many false positives and inaccurate with inconsistent annotations. In addition, each available PPI database uses its own extraction, curation and storage protocols, they do not necessarily explore the same scientific papers and are composed of different compositions of experimentally and computationally determined interactions. Prieto and De Las Rivas [41] have shown a limited intersection and overlap between the six major PPI databases for human proteins (BioGRID, BIND, MINT, HPRD, IntAct, DIP) [42]–[47]. As the information contained in these databases is partly complementary, knowledge on the interactome can be increased and improved by combining multiple databases. Notably, even the union of all databases is still incomplete with many unknown components and pathways, reaching coverage of 31% of the human proteome, corresponding to 12053 proteins and 83670 interactions. The five largest databases are considered in this contribution, with HPRD containing 63.3% of the known PPIs, BioGRID 40.9%, IntAct 34.9%, MINT 22.5%, and BIND 9.7% according to data in 2008 (see the ‘statistics’ section on http://bioinfow.dep.usal.es/apid/, [41]). We did not consider DIP as this database only contains 1.75% of the known PPIs [47]. Four of the largest databases (BIND, HPRD, MINT and IntAct) are combined with high-throughput experiments in the Online Predicted Human Interaction Database (OPHID), a catalog of 60675 known human PPIs [25]. This manually-curated, literature-derived catalog was further expanded with predictions for 34824 interactions that occur in other model organisms but with both human orthologs conserved in humans. In this way, OPHID could extend the human interactome with a set of proteins that have not yet been included in literature-based databases. We translated all biomolecules included in OPHID to one or multiple corresponding genes, after which relations between the resulting genes were extracted. The same definition for gene pairs was used for the remaining PPI database BioGRID [42]. As this database is multi-organismal, we only extracted the interactions between human genes. The list of gene pairs for this database was much smaller than for OPHID because the conversion from protein to gene had already been done by its curators.

STRING [48], the Search Tool for Recurring Instances of Neighboring Genes, is extracted from PPI networks and predictions based on comparative genomics and text-mining. It lists potential functionally associated proteins based on the genomic association of their genes. It thereby acts as a metadatabase that maps all interaction evidence onto a common set of genomes and proteins, by weighting and integrating information from numerous sources: PPI databases, high-throughput experimental interaction data, associations highlighted in published literature, interaction transfer between organisms, functional co-expression of genes, conserved neighborhood, gene fusions and phylogenetic co-occurrences. All resulting individual scores are integrated in a combined score, reflecting the confidence for each predicted protein association. This results in an increased confidence when an association is supported by multiple types of evidence. We truncated the interaction network to a stringency of 500 for the confidence score and only retained gene interactions that were experimentally determined.

#### Domain-domain interactions

As often only a fraction of a protein directly interacts with its biological partners, we also investigated the use of inter-chain DDIs, that is, interactions between domains from different proteins. To increase coverage and quality, we considered two comprehensive resources that have collated all known and predicted DDIs from various sources. The database DOMINE [49] and the Unified Domain Interaction database (UniDomInt) [50], both using Pfam-A domain definitions, combine DDIs based on experimentally derived 3-dimensional structures in the Protein Data Bank and DDIs predicted by 8 and 9 computational approaches, respectively. In the DOMINE database [49], each predicted DDI is given a discrete confidence level high, medium or low. We did not include the low confident DDIs that were only predicted by one computational method, reducing the number of DDIs to 6689. UniDomInt [50], on the other hand, provides a continuous reliability score between 0 and 1. We set the minimal threshold to 0.1, including 8470 DDIs. Genes of which the proteins interact via a domain-domain interaction were linked in the corresponding graph representations.

#### Protein families and domains

To cover an as broad as possible range of gene-related information, we additionally investigated whether incorporating information on proteins belonging to the same protein family or sharing the same protein domain improves classification. Information on the structural and functional properties of proteins was extracted from the databases PROSITE [51] and Pfam [52]. PROSITE is a database that uses amino acid patterns and profiles for the identification of protein families, domains and functional sites. The raw data provided by KEGG were used to extract gene pairs with one or multiple protein domains, families or functional sites in common. Pfam [52] is another large comprehensive and accurate collection of protein domains and families. As a similar strategy was followed as for PROSITE, no distinction could be made between the manually curated Pfam A families and the automatically generated Pfam B families. Also for Pfam, gene pairs were defined as genes of which the proteins belong to the same protein family or share the same protein domain.

#### Transcription factors

Finally, microRNAs (or miRNAs) are a class of small non-coding RNA species with critical functions across various biological processes by regulating gene expression. Evidence has suggested that miRNAs may play a role in human cancers [32]. We therefore defined gene pairs based on the miRNAs by which they are regulated, using four available microRNA databases. The miRBase (microRNA database) [53] contains a pipeline for predicting miRNA target genes in mRNA sequences based on the miRanda algorithm. P-values were assigned to individual miRNA-target binding sites, connecting each miRNA to a list of predicted gene targets. For the 851 miRNAs included in the database, we set the p-value to 0.001 to include only the most confident predicted miRNA-target assignments. MiRNAmap [54] is another database containing 470 miRNAs, dividable into known miRNA genes obtained from miRBase and putative miRNA genes identified by comparative sequence analysis. Besides experimentally verified miRNA targets obtained from both specialist literature and TarBase [55], three computational tools miRanda, RNAhybrid and TargetScan were used for the identification of putative miRNA targets. To reduce the rate of false positive target site predictions, we applied the three criteria that were proposed by Hsu and colleagues [54]: target sites must be predicted by at least two tools, they must be located in accessible regions, and the target genes must contain multiple target sites. Next, microRNA.org [56] contains target predictions also based on the miRanda algorithm with miRNA sequences obtained from miRBase, but the predicted target sites of 677 miRNAs were filtered according to evolutionary conservation of sequence blocks across multiple vertebrates. We set the threshold for the conservation score to 0.70 to select target sites that are conserved in mammals. Finally, the target site prediction tool TargetScan [57] provides a p-score, corresponding to a Bayesian estimate of the probability that a site is conserved due to miRNA targeting. These scores reflect the biological relevance and efficacy of each site. We set the threshold to 0.3, corresponding to 74 miRNA genes. For all these databases, gene pairs were defined as genes targeted by the same miRNA.

The programming language Perl was used for the conversion of each secondary data source into a list of gene pairs, used as input for the calculation of the pseudoinverse Laplacian.

### Pseudoinverse Laplacian

Many biological processes are representable as a large-scale sparse network. Each secondary data source, and more specifically the list of extracted gene pairs, can therefore be represented as a weighted, undirected graph with symmetric weights 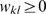, assigned to each edge between a pair of different nodes *k* and *l*. Such a graph is composed of *p* nodes representing the genes, and the edges connect genes that are linked with regard to the secondary data source under study. The graph is characterized by a weighted adjacency matrix *W* = [*w_kl_*], *k,l* = 1..*p*, and the diagonal degree matrix *D* with degrees *d_1_* to *d_p_* as diagonal elements. The degree of a node *k* is defined as the sum of the weights *w_kl_* for node *k* across all nodes *l*. From spectral graph theory, we can now define the unnormalized graph Laplacian matrix *L* as the difference between the degree matrix and the weighted adjacency matrix (*L* = *D*−*W*). This matrix is symmetric and positive semidefinite. Genes belonging to similar pathways will be connected by a relatively large number of short paths, while fewer, typically longer paths connect genes with completely separate functions. Finally, as our aim is to add extra gene-related information with respect to the traditional similarity measure rather than to discard genes for which no information is available, self-loops were added to isolated genes, setting their degree to 1.

Fouss and colleagues [30] have shown that the Moore-Penrose pseudoinverse *L^+^* of the Laplacian matrix of a graph can be interpreted in terms of similarity between pairs of genes in the interacting network. Matrix entries increase when the number of paths connecting two nodes increases and when the length of the paths decreases. In this manuscript, we will present the *p x p* pseudoinverse Laplacian matrix *L^+^* of the graph corresponding to secondary data source *m* as 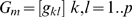. The graph of each secondary data source and the extraction of *G_m_* are presented as step 1 in Figure 2. In case of a fully connected graph, the *G*-matrix is expected to be fully dense. Isolated genes, however, introduce zero patterns in the inverse, thereby reducing the density as illustrated in Table 3. Other graph kernels such as the diffusion kernel [58] are less suitable since they require an extra parameter to be optimized. Also, instead of the unnormalized Laplacian, the normalized version *D^−1/2^LD^−1/2^* in which the connectivity of each gene is taken into account can be considered as well. However, the use of the normalized Laplacian did not lead to an improvement in performance (results not shown).

### Kernel methods and weighted Least Squares Support Vector Machines

Kernel methods are a group of algorithms that can handle a very wide range of data types such as vectors, sequences and networks. They map the data *x* from the original input space to a high dimensional feature space with the mapping function Φ(*x*). This embedding into the feature space is performed by a kernel function *K*(*x^i^*,*x^j^*). This function efficiently computes the inner product 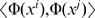 between all pairs of data items *x^i^* and *x^j^* in the feature space, resulting in the kernel matrix. The size of this matrix is determined only by the number of data items, whatever the nature or the complexity of these items. For example, a set of 100 patients each characterized by 5000 gene expression values is still represented by a 100 *x* 100 kernel matrix [59]. The representation of all data sets by this real-valued square matrix, independent of the nature or complexity of the data to be analyzed, makes kernel methods ideally positioned for heterogeneous data integration.

A kernel algorithm for supervised classification is the Support Vector Machine (SVM) developed by Vapnik [60] and others. Contrary to most other classification methods and due to the way data are represented through kernels, SVMs can tackle high dimensional data (for example microarray data). Given a training set 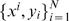 of N samples with feature vectors 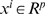 and output labels 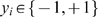, the SVM forms a linear decision boundary in the feature space 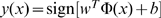 with maximum margin between samples of the two considered classes, with *w* representing the weights for the data items in the feature space and *b* the bias term. This corresponds to a non-linear discriminant function in the original input space. A modified version of SVM, the Least Squares Support Vector Machine (LS-SVM), was developed by Suykens and colleagues [27]–[28]. On high dimensional data sets, this modified version is much faster for classification because a linear system of equations instead of a quadratic programming problem needs to be solved. The constrained optimization problem for an LS-SVM has the following form: 
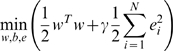
 subject to 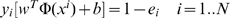 with *e_i_* the error variables tolerating misclassifications in case of overlapping distributions, and *γ* the regularization parameter which allows tackling the problem of overfitting. It has been shown that regularization is very important when applying classification methods to high dimensional data, even for linear classifiers [61].

In many two-class problems, data sets are skewed in favor of one class such that the contribution to the performance assessment criterion of false negative and false positive errors is not balanced. We therefore used a weighted LS-SVM in which a different weight *ζ_i_* is given to positive and negative samples, in order to account for the unbalance in the data set [29]. The objective function changes into: 
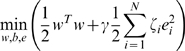
 with 
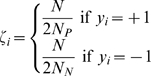
 and *N_P_* and *N_N_* representing the number of positive and negative samples, respectively.

### Adapted kernel function

Any symmetric, positive semidefinite function is a valid kernel function, resulting in many possible kernels - for example linear, polynomial, and diffusion kernels. They all correspond to a different transformation of the data, meaning that they extract a specific type of information from the data set. In this paper, the linear kernel function was investigated. Traditionally, a kernel matrix based on a linear kernel function is represented as 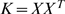 with 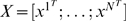 the *N x p* patient microarray data, *N* the number of samples, *p* the number of measured genes (here, reduced to the 5000 most varying genes), and ^T^ representing the transpose of a vector or matrix. In patient domain, each matrix entry *K_ij_* corresponds to 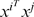, with *x^i^* and *x^j^* the gene expression profiles of samples *i* and *j*, respectively. We incorporated a secondary *G*-matrix (introduced in the section on the pseudoinverse Laplacian) in our kernel-based classification framework by expanding the kernel matrix 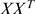 to 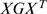 (step 2 in Figure 2). Each entry in this expanded kernel matrix now corresponds to 
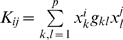
. Each *G*-matrix thus exhaustively relates the gene expression profiles of patients, weighted by its entries *g_kl_*. As each secondary data source leads to a different kernel matrix, normalization is required to make them comparable. The normalized kernel matrix 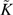 was therefore considered, defined as 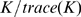 with *trace(K)* the sum of the diagonal elements of *K*.

### Combining classifiers

It is not known beforehand which secondary data sources are relevant for the problem at hand and thus which of the sources will increase prediction accuracy. The predictions of the LS-SVM models, each built with the inclusion of one secondary data source, were therefore combined at a second level (step 5 in Figure 2). Duin and Tax [62] have shown that no combining rule is optimal in multiple combination tasks. We therefore considered multiple combination schemes that have proven to be useful in specialist literature and that provide continuous predictions on which the AUC can be calculated. A comparison of AUC values is less sensitive to the specific cut-off level used for assigning observations to different classes.

A distinction is made between fixed and trained combining rules [62]–[63]. As fixed combining rules, we investigated mean and median. Although the mean or sum rule assumes independent classifiers, it may also work for similar classifiers with independent noise behavior, thereby reducing the error on the estimated output values. The same holds for the median rule, likely to yield more robust results in comparison to the mean rule. Fixed combining rules, however, are almost always suboptimal while a trainable combiner may lead to a more significant improvement with respect to static combiners [63]. Moreover, statistically independent classifiers are not required when a linear or non-linear combiner is trained [64]. Although requiring additional data and at the cost of additional training, a weighted sum of the predictions of the individual classifiers was considered. The weights were set to the raw training AUC values or to these values after scaling to the half-open interval ]0,1]. Furthermore, an exhaustive search among all possible unweighted combinations of individual classifiers was performed. We additionally considered three more advanced models, being naïve Bayes (NB), logistic regression (LR) and linear discriminant analysis (LDA). Both NB and LDA are ideal methods when the number of training observations is limited. Although NB is based on the assumption of independence between predictors, a good performance has been shown for functional dependencies, that is, predictors that are generated from the same underlying distribution [65]. This is likely to be the case in this set-up as the latent variables are obtained from the same microarray data set, modified by a specific secondary data source.

### Model building

In this study, each data set was split into 10 folds, stratified to outcome. 50% of the data corresponding to folds 1 to 5 was used for training the individual classifiers. This part of the data was normalized per gene, and the obtained gene characteristics were used for normalization of folds 6 to 10. An internal 5-fold cross-validation on folds 1 to 5 was used for the optimization of the regularization parameter *γ*. Forty possible values for *γ* ranging from 10^−4^ to 10^6^ were considered on a logarithmic scale. The final model parameter was chosen corresponding to the model with the highest AUC. In case multiple models had the same AUC, the model with the lowest balanced error rate and an as high as possible sum of sensitivity and specificity was chosen. An LS-SVM model was rebuilt on the entire training data with the optimal regularization parameter and applied to the remaining 50% of the data. Similar results were obtained when the L-curve was used for the selection of *γ* [66]. As the *γ* values of the individual LS-SVM models were checked cautiously to prevent overfitting and to assure good generalization performance, the combining rules were learnt on 80% of the data of which 30% (that is, folds 6 to 8) were new with respect to the first training phase. Considering 80% of the data in the second phase for training a combined classifier has the extra advantage that the peaking phenomenon is countered, defined as the decrease in classification accuracy when too many features are included in the classifier [67]. Among the considered combined classifiers, especially LDA suffers from this phenomenon [68]. LDA is also poorly posed when the number of observations and parameters to be estimated is comparable [69]. The use of 80% of the data guarantees that the number of training observations sufficiently exceeds the number of individual classifiers. The last 2 folds corresponding to 20% of the data were used for validation. Not only the combined classifiers but also the baseline LS-SVM model built only on the microarray data and the models with the individual use of each of the secondary data sources were validated on the same observations. To reduce the random variation in the selection of training and test data, the split of the data into 10 folds was repeated 200 times. A comparison of the AUC values was performed between the baseline model and all other models using the one-sided paired-sampled t-test. However, the overlap in training and test set between the 200 experiments can increase the probability of type I errors (that is, rejecting a true null hypothesis). Moreover, we are not only interested in the performance for a specific problem, but rather in the general performance on multiple data sets. The obtained results per data set were therefore confirmed by repeating the comparisons over all data sets with the average performance per set, using the non-parametric Wilcoxon signed-ranks test [70].

Out of the 16 microarray data sets, 10 were randomly selected for training indicated with the symbol *T* in Table 2. These 10 data sets were used to determine the secondary data sources that improved performance compared to the baseline model in the majority of data sets. These data sets were subsequently used to define which of the combination rules performed best. The set of the best rules was applied to 6 validation microarray data sets indicated with the symbol *V* in Table 2.

To assess the dependency between individual LS-SVM models, we considered mutual information (MI) [71]. When two random variables *x* and *y* have probability distributions *P(x)* and *Q(y)*, mutual information *I* is defined as the relative entropy between the joint distribution *R(x,y)* and the product distribution *P(x)Q(y)*: 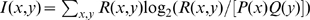. Mutual information is a measure of the reduction in uncertainty about one variable given the other, meaning that variables are statistically independent when MI = 0. A higher MI indicates that the two variables are non-randomly associated with each other [71]. In this study, the variables *x* and *y* are binary and defined as 1 with the classifier predicting a sample being of the positive class and 0 otherwise.
